# A pre- and post-intervention study of infection control in equine hospitals in Sweden

**DOI:** 10.1186/s13028-014-0052-4

**Published:** 2014-08-22

**Authors:** Karin Bergström, Ulrika Grönlund

**Affiliations:** 1 Department of Animal Health and Antimicrobial Strategies, National Veterinary Institute, Uppsala, SE 750 89, Sweden; 2 Department of Clinical Studies, Swedish University of Agricultural Sciences, Uppsala, SE 750 07, Sweden

**Keywords:** Infection control, Horses, Equine hospital, Intervention, Compliance

## Abstract

**Background:**

Detection of nosocomial methicillin-resistant *Staphylococcus aureus* infections in horses in Sweden has increased attention on infection control (IC) in equine hospitals. This study established baseline data on IC programmes within such settings, evaluated compliance with some IC procedures before and after an education intervention, and examined barriers to compliance.

The study was carried out between 2008 and 2011 in four Swedish equine hospitals. Data on current IC of each hospital, purchase data on hand sanitisers and disposable gloves per patient, and direct observations of compliance with procedures were monitored pre- and post-intervention. The intervention comprised a lecture on common IC and a review of each hospital’s current procedures. For comparison, retrospective purchase data were reviewed. A questionnaire on individual compliance, experiences and opinions of IC was issued to employees.

**Results:**

Three hospitals completed the study, while the fourth reported its IC procedures and completed the questionnaire. Actual numbers of procedures, content and level of documentation differed among the hospitals. Similarities were poor or absent IC implementation strategy, lack of active surveillance of compliance with procedures and no monitoring of such as nosocomial infections. Among the hospitals which completed the study, two reported pre-intervention observation of compliance, while all three reported post-intervention observations. The purchase data showed trends for changes over time, although not uniformly related to the intervention. One hospital demonstrated a significant post-intervention increase in compliance with glove procedures, accompanied by a non-significant post-intervention increase in purchases figures. Compliance with dress code and personal appearance was high in all three hospitals (92-100%), while compliance with hand hygiene procedures was generally poorer. Barriers to compliance cited in the questionnaire (data from four hospitals) included insufficient supplies of hygiene products, lack of readily accessible places for cleaning, insufficient knowledge and high workload.

**Conclusions:**

Potential for easily attainable improvements in IC, such as traceability of documents, implementation strategies and surveillance of efficacy, was revealed. Attention to hand hygiene implementation and improvement of logistics appeared important. Data on purchases per patient were readily available and therefore applicable for intra-hospital surveillance of IC trends over time.

## Background

Nosocomial infections are a hazardous problem in human and veterinary medicine. Zoonoses and antimicrobial-resistant bacteria aggravate the problem [1]-[6]. Nosocomial infections with methicillin-resistant *Staphylococcus aureus* (MRSA) and *Salmonella* spp. have been reported in hospitalised horses and pose a risk to other animals, but also to humans [2],[3],[5],[7]-[9]. Veterinary healthcare maintains high standards, but to become excellent the risk of nosocomial infections cannot be ignored [10],[11]. Veterinarians also have ethical and legal responsibilities to prevent the spread of infectious diseases among animals. Furthermore, employers must take responsibility for minimising the spread of zoonotic agents in animal hospitals.

In Sweden, the first cases of MRSA infections detected in horses, which occurred in an outbreak of surgical site infections within an equine hospital, increased awareness of the need for infection control (IC) in equine medicine [12]. Based on knowledge of human medicine IC, procedures were implemented in the hospital to curb the outbreak [13]. Descriptive studies of infection prevention and control interventions in equine hospitals, as well as expert guidance, strategies and advice, have been published [2],[5],[8],[11],[14],[15]. However, the implementation, compliance and effectiveness of IC procedures adapted to equine species and employees in equine hospitals have been less well studied.

The aims of this study were to establish baseline data on IC programmes in Swedish equine hospitals, evaluate compliance with IC procedures before and after an intervention, and examine barriers to compliance.

## Materials and methods

### Study design

This was a descriptive pre- and post-intervention study using measures to monitor compliance with IC procedures and barriers to compliance.

### Settings

Three equine hospitals (A, B and C) and one clinic (D), hereafter named hospitals A-D, voluntarily joined the study, which was carried out between January 2008 and May 2011, with hospital A starting about a year prior to the others. During the study period, hospitals A, B and C provided stationary care and performed elective and emergency surgery. In addition, hospital A is a teaching hospital and thus in addition to employees, veterinary students and teaching staff also use the facilities. During the study period for hospital A (January 2008-May 2010), it received approximately 6500 visits and 470 surgery patients per year. Hospital B reported approximately 4600 visits and 450 surgery patients, and hospital C 4800 visits and 520 surgery patients per year during the study period (January 2009-March 2011). Hospital D did not report these data.

The study design and requirements were explained to the hospital managers and they were asked to appoint one or two staff members as contacts and observers for the study on-site. These contact individuals had to perform observations to monitor compliance with procedures, report purchase figures etc. Protocols used in the study were developed in agreement with the hospital managers and the contact individuals.

### Intervention

The intervention applied comprised a lecture on general IC theory and practice, especially basic hygiene, together with a review and active discussion of the hospital’s current procedures. A practical demonstration on how to disinfect hands, using a fluorescent agent and UV-lighting to visualise correct/incorrect disinfection, was given on the intervention occasion (HandCheck^R^, Ecozen, The Ecowest Group, Gothenburg, Sweden). In human and veterinary health care, hand hygiene procedure includes washing of visibly dirty hands, followed by hand sanitising. If the hands are not visibly soiled, hand sanitising is considered enough. In general hand hygiene is carried out prior to operations and between patients.

### Monitoring

The objective data monitored were: purchase figures, number of visiting patients, days of stationary care and current IC procedures. Data on purchase totals for hand sanitisers and disposable gloves were converted to purchases per patient and per patient-day. These data were gathered pre- and post-intervention and also retrospectively, i.e. prior to the intervention monitoring.

The subjective data comprised direct, anonymous observations (at personal level) of compliance with some specified IC procedures, adapted to the current IC programme in each setting, e.g. dress code, hand hygiene and glove use (Table 1). These observations were performed pre- and post-intervention, in daytime during ordinary working hours, and separately in stationary and day patient wards. Prior to the start of audits, the individuals responsible underwent a review of the existing IC procedures, combined with an introduction and short training session on the study protocols. The definition of non-compliance with a procedure included that procedure had not being carried out at all, but also being performed incorrectly in relation to IC procedures No distinction was made between these two forms of non-compliance in the protocol. A copy of the observation protocols used is available upon request from the first author.

**Table 1:** Infection control procedures in equine hospitals before/after an intervention

**Hospital**	**A**	**B**	**C**	**D**
Dress code^1^	+/+^7^	+/+	−^7^/+	+/0^7^
Personal appearance^2^	+/+	+/+	−/+	+/0
Hand hygiene^3^	+/+	+/+	−/+	+/0
Disposable gloves^4^	+/+	+/+	+^6^/+	+/0
Cleaning regulations	+/+	+/+	−/+	+/0
Extended^5^	+/+	+/+	−/−	-/0
Reading and signing the procedures	+/+	+/+	−/−	-/0

^1^Short-sleeved working clothes, except in cold weather, changed regularly or if dirty.

^2^No rings/wrist watches, short nails without polish, long hair tied back, etc.

^3^Hand washing of visibly dirty hands, otherwise hand disinfection, prior to operations and between patients.

^4^Gloves used at risk of contamination, handling dressings and wounds, inserting catheters etc.

^5^Additional procedures, such as cleaning/disinfection of instruments etc.

^6^Non-documented procedure for glove use, split in two, at insertion of IV catheters and in wound treatment.

^7^+ = routine established, − = not established, 0 = not reported.

A voluntary and anonymous (at person level) questionnaire was issued to staff on-site at the intervention seminar and they were asked to complete it and return it immediately. The questionnaire contained two closed and two open questions concerning the employee’s individual compliance, knowledge, experiences and opinions on hospital IC procedures. The responses were categorised into groups and statements with similar meaning were quantified. A copy of the questionnaire is available upon request from the first author.

The current IC procedures and their documentation were reviewed together with the manager at each hospital. Decisions on whether and how their procedures could be revised for continuous improvement of IC were made after the intervention.

### Statistics

Descriptive statistics on purchases per patient and patient-day were calculated for each hospital. The differences in pre- and post-intervention observations were investigated using Fisher’s exact test. The questionnaire was summarised using descriptive statistics.

## Results

Hospital D reported pre-intervention IC procedures, joined the intervention and answered the questionnaire, but thereafter reported no data.

### Infection control

The pre- and post-intervention IC procedures used at the hospitals are shown in Table 1. The procedures in use at hospitals A and B had been written with support from human IC expertise, had been distributed to all personnel to read and sign and were available on the internal website. Apart from this ‘read and sign’ practice, none of the hospitals had any plan for implementation. In hospital A, the documents were traceable to the extent that they were annotated with date and revision number and signed by the relevant member of staff. Post-intervention documents, new and/or revised, were consequently made traceable at all three hospitals.

Monitoring of compliance with IC procedures and/or active surveillance of IC efficacy was not currently included in the IC programme of any of the hospitals.

### Questionnaire

The questionnaire was answered by 56%, or 44 out of 78, of participating staff at all four hospitals (hospital A: 21/36, B: 10/21, C: 2/10 and D: 11/11). In all, 86% (38/44) of respondents stated that they were fully or partly familiar with the content of their hospital’s IC procedures and 6/44 that they did not know any of the content. Furthermore, 82% (36/44) complied fully or partly with the procedures, 5/44 did not comply at all and 3/44 did not give any answer. At hospitals A and B, all respondents answered that they complied fully or partly. Responses to the two open questions are shown in Table 2. To summarise, among the perceived barriers to compliance, practical reasons were most commonly cited. The other two were insufficient knowledge of IC procedures and heavy or acute workload.

**Table 2:** Perceptions of infection control among respondents at four equine hospitals (no. of answers)

**Question 1: If you comply only partly or not at all with IC procedures, what is/are the reason/s for this?**		
***Practical reasons***	***Knowledge***	***Str0065ss/lack of awareness***
• Short sleeves etc., impractical when cold (n = 3)	• Have not read any procedures (4)	• Too many cases at one time (1)
• Insufficient supply of hygiene products (2)	• Don’t know of any/there are no procedures (2)	• Acute case, no time for hygiene (1)
• No place to wash shoes/boots (2)	• Lack of information (1)	• Oblivion or no time when work is stressful (3)
• Theory is one thing, must work in practice, tedious (3)	• Lack of education (1)	• Reflex, routine (1)
• Ongoing rebuilding, difficult (1)		
**Question 2: Enter, in your opinion, the most important reason/reasons for applying hygiene procedures in an animal hospital.**		
***Contagious risk/protection***	***Antimicrobial resistance***	***Other***
• Reduce/prevent spread of infectious agents to humans and animals (n = 38)	• Counteract spread of resistant agents (5)	• Good for customer (2)
• Provide best possible care (4)	• Reduce use of antimicrobials (1)	• Reduce costs (1)
• Fewer complications (1)		• Confidence (1)
• Prevent what cannot be seen (1)		• Nice with clean working environment (2)
• Staff security (1)		• Box to tick “There is no reason” (0)

### Purchase figures and observations

Hospitals A, B and C reported their objective purchase figures for hand sanitisers and disposable gloves and number of patients, retrospectively and pre- and post-intervention, as requested. However, data allowing conversion to purchases per patient-day were only reported by hospital B (Table 3). The figures presented in tables should only be compared within each hospital over time, not between hospitals, as the total of employees differed and one hospital also had students and teachers using the facilities.

**Table 3:** Purchase figures on hygiene products per patient (per patient-day) monitored at equine hospitals

	**Retrospective**^**1**^	**Pre-intervention**^**2**^	**Post-intervention**^**3**^
**Hospital A**			
Months of recording	***10***	***6***	***12***
Hand sanitisers, ml	82	48	50
Pairs of gloves	33	20	19
**Hospital B**			
Months of recording	***8***	***6***	***12***
Hand sanitisers, ml	3 (2)	6 (4)	8 (5)
Pairs of gloves/patient	7 (5)	13 (8)	13 (9)
**Hospital C**			
Months of recording	***11***	***5***	***12***
Hand sanitisers, ml	3	4	6
Pairs of gloves	2	2	7

^1^Retrospective figures prior to the actual start of the study.

^2^Figures prior to an intervention, which included education of infection control procedures.

^3^Figures after the intervention.

The pre-intervention observations were not carried out according to plan, shown in Table 4. As the observation protocol was adapted to each hospital’s IC programme, the selection of procedures included in observations differed. Observations of compliance were perceived as time-consuming, interfering with daily work.

**Table 4:** Rate in% (no. of observations) of pre- and post-intervention compliance with procedures in equine hospitals

**Procedure**	**Period**^**1**^	**Ward**^**2**^	**Hospital A**	**B**	**C**
Dress code	Pre	S	92 (96)	ND	NA^4^
		D	NA^4^	ND	NA
	Post	S	100 (226)	99.6 (231)	100 (35)
		D	100 (92)	99.6 (255)	100 (50)
		NS	--	--	100 (50)
Personal appearance	Pre	S	100 (96)	ND	NA
		D	ND	ND	NA
	Post	S	100 (226)	99.1 (231)	NA
		D	100 (92)	98.0 (251)	NA
Hand hygiene^3^	Pre	S	9.6 (96)	ND	NA
		D	ND	ND	NA
	Post	S	ND	99.6 (231)	37 (35)
		D	32 (92)	87.1 (256)	24 (50)
		NS	--	--	22 (59)
Hand washing^3^	Pre	S	ND	NA	NA
		D	100 (2)	NA	NA
	Post	S	100 (26)	NA	NA
		D	100 (49)	NA	NA
Glove use	Pre	S	ND	ND	^5^
		D	ND	ND	
	Post	S	ND	96.4 (229)	
		D	ND	98.4 (190)	

^1^‘Pre’ denotes pre-intervention observations, ‘Post’ post-intervention observations.

^2^S = stationary ward, D = day patient ward, NS = not specified.

^3^Hand hygiene was equal to hand washing and/or sanitising in hospitals B and C. In hospital A it was split into two procedures, hand sanitising (in the table = hand hygiene) and hand washing.

^4^ND = not performed, or observation interpretation was not according to procedure, NA = not applicable, due to lack of procedure.

^5^Hospital C, glove use see Results, Purchase figures and observations.

#### Hospital A

The retrospective purchase figures were markedly higher than both the pre- and post-intervention figures, while no change in purchase figures per patient was evident after the intervention in hospital A (Table 3).

Where an adequate quantity of data was reported and calculations were possible, compliance with procedures showed very little or no difference before and after the intervention (Table 4). Their hand hygiene procedure was divided into two sets of observations, hand washing (soap and water) and hand sanitising (alcohol rub). Compliance with the hand washing procedure was absolute, while compliance with the hand sanitising procedure was poor. Observation data of the glove use procedure were excluded, as the observer had interpreted the procedure different over time.

#### Hospital B

Hospital B had a pre-intervention increase in purchase figures per patient. Their data also made it possible to calculate figures per patient-day. These two ways of calculating the data gave similar trends over time (Table 3).

Hospital B reported the highest compliance with hand hygiene procedure (Table 4), but only post-intervention observations were performed and comparison of pre- and post-intervention procedures was not possible.

#### Hospital C

In hospital C, a non-documented procedure for glove use (Table 1): (i) on insertion of IV catheters and (ii) in wound treatment was the only measurable procedure either pre- or post-intervention. After the intervention, there was an increasing trend in glove purchase figures (Table 3), associated with a significant increase in compliance with the glove use procedure from 76 to 89% (*P* = 0.015).

Compliance with the glove use procedure when inserting IV catheters in the stationary ward had an post-intervention increase from 57% (31/54) to 93% (39/42) (*P* < 0.001). In the day ward, insertion of IV catheters was rarely done and the data excluded. Compliance with the procedure in wound treatment in the stationary ward increased non-significantly from 92% (43/47) to 94% (44/47) post-intervention, while in the day ward there was a non-significant decrease, from 93% (14/15) to 73% (19/26) post-intervention.

## Discussion

The emergence of antimicrobial-resistant bacteria in animals calls for reinforced implementation of IC in veterinary healthcare. To our knowledge, this is the first study in equine hospitals to establish baseline data on current IC programmes, monitor pre- and post-intervention compliance with procedures and explore staff’s perceptions of IC and of the barriers to compliance. Taking part in a study like this probably inspires further improvement of IC with hospitals, which in itself is a positive outcome.

Documentation and traceability of procedures markedly improved in the hospitals during the study. Dated and revision-numbered documents signed by a member of staff with specific responsibility seem an obvious first requirement in any quality assurance system. Moreover, written information stays the same, while oral information can change considerably over time, so a reminder seems warranted for this easily implemented quality improvement.

When procedures are in order, compliance has to follow. A logical start to achieving compliance is provision of information and education about IC and allocation of time for staff to read and learn about the procedures [16]. Low awareness of IC procedures was stated in some responses to our questionnaire, which indicates that provision of information on the written procedures needs more attention. The practice of staff signing the documents after reading them applied in two of the hospitals was an approach to check that everyone had been informed and to aim at compliance.

The education intervention applied in this study was another approach to improve compliance. However, only one of the three hospitals concerned showed a significant increase in compliance post-intervention, regarding their glove use procedure. This despite that 84% (38/44) of the staff in the questionnaire stated their awareness of IC, by responding that IC procedures reduce or prevent the spread of animal and human infectious agents. The hospital reporting the post-intervention increase had no written procedures prior to our intervention, indicating a potential for improvement and thereby a possible impact of the education. Significant rise (21-42%), in correctly performed hand hygiene after education in a small animal veterinary teaching hospital has been reported previously [17]. Moreover, insufficient education has been reported as a cause of low compliance with hand hygiene in small animal practices [18]. However, conflicting results have been reported in another veterinary study, where no improvement in hand hygiene occurred after an education campaign [19]. This was indicated also by hospital A and B’s purchase figures, which were approximately the same pre- and post-intervention. Comparing results among the studies have inherent setbacks, as education was not standardised, the pre-conditions differed etc.

Hospital A, with a clear decline in the pre- and post-intervention figures compared with retrospective purchase data (Table 3), had experienced an outbreak of MRSA a few months before the retrospective data were collected [12], and it is likely that this outbreak was a stronger trigger for revising and improving IC than our intervention. The differences in response after the intervention in this study signify a central element in IC, namely implementation processes. Identifying reasons for hospital A’s declining purchases, and thereby suspected declining compliance some time after the outbreak, as well as triggers for increased compliance would be useful in developing tools for efficient implementation and continuous compliance. The observed discrepancy in compliance with different procedures, e.g. low compliance with hand hygiene and excellent compliance with dress code and personal appearance also point at mixed success of implementation. Compliance with hand hygiene procedures seems similarly low in other veterinary hospitals, according to the few studies found. Three studies, performed in small animal hospitals, reported between 21 and 42% compliance [17]-[19]. A human healthcare review of 96 published studies on the subject reported a median hand hygiene compliance rate of 40% and shows that compliance with hand hygiene procedures is low even there [20]. The common poor compliance with hand hygiene enhances the importance of strong efforts to succeed with implementation.

To obtain more information on hand hygiene compliance, hand washing (soap + water) and hand sanitising (alcohol rub) were observed as two separate procedures in one hospital. A difference was observed, with absolute compliance with hand washing and poor compliance with hand sanitising. According to the procedures, hand washing was required in cases of visibly dirty hands and had to be followed by use of hand sanitiser. A plausible explanation for the discrepancy could be that hand washing was habitual, as handling of horses causes dirty hands, whereas use of hand sanitiser was a more recent practice and presumably had lower awareness. Incorrectly performed hand hygiene procedures could also be a reason for the low compliance figures, as the definition of non-compliance we used included ‘not performed at all’ and ‘incorrectly performed’. Thus, observation protocols should make a distinction between the two. Knowledge of differences between these would be helpful in devising training strategies.

The strong compliance with dress code and personal appearance was interesting, as successful implementation also provides knowledge. As non-compliance is visible to all, it is likely that the staff felt forced to comply. Furthermore, this once-daily habitual procedure presumably required less effort of memory or action than the hand hygiene procedure.

The most commonly cited barrier to compliance in the questionnaire was practical reasons, e.g. insufficient supply of hand sanitiser or lack of accessible places to wash equipment. A previous study of 18 small animal hospitals found that hand soap was more available (85%) than alcohol-based hand sanitiser (12%) [18]. Lack of readily available hand rub has been correlated to lower compliance also in human hospitals [21],[22]. Knowledge and understanding of barriers to compliance with IC procedures are an important element in the implementation process. The questionnaire we used was a straightforward tool for identifying barriers to compliance and most causes expressed in the responses, such as unavailability and unsatisfactory supply of cleaning products, could be easily remedied by managers interested in improving IC compliance. Questionnaires could be repeated to monitor improvements and failures and obtain new information on barriers.

Monitoring of compliance includes mainly hand hygiene and has been measured for some time within human medicine. Three different methods are used: (i) observation of compliance, (ii) measurement of hand sanitiser (purchase or usage) and (iii) self-assessment [23]-[26]. Surveillance provides information on a programme’s function, but prior to this study the participants did not monitor compliance with procedures, nor did they actively monitor such as occurrence of nosocomial infections. Undetected weaknesses could result in misplaced trust in the IC, meaning a risk to patients and a waste of money in case of ineffective procedures. Therefore, when planning IC programmes, surveillance should be a part of the process [10],[11],[26],[27]. The concurrent increase in objective (purchase) and subjective (observations) figures in hospital C strengthened the credibility of both sets of data as measures of compliance with glove use procedures in equine hospitals. In human healthcare, associations between observations of hand hygiene and purchase quantities of sanitisers have been found in 10 of 13 clinics [24].

Recording data on purchase figures and patient numbers was perceived to be uncomplicated and straightforward by the hospitals in this study and thereby appealing as a tool for long-term monitoring of trends. The actual figures would most likely not be subject to bias, but converting to purchase per patient raises the possibility for bias, as the time patients spend in hospital differs. However, the comparison of data on purchases per patient and purchases per patient-day in this study, showed similar trends for the two. This suggests that continuous monitoring of purchases per patient could be of value for intra-hospital trend analyses, but this finding should be verified by more data. Other bias factors to consider are the number of employees, purchasing routine (buying monthly, quarterly etc.) and whether sanitisers are used for other than the intended purpose. In a stable group of employees, the influence of these bias sources would be negligible over time and by linking inventory figures on storage to purchase data, more reliable running figures could be obtained. Incorrect use could be controlled by detailed observations. Purchase data could be converted to a rough estimate of number of hand sanitising occasions in a setting, if the precise amount of sanitiser used per occasion is defined, but it was not made in this study. A more exact and comprehensive, but costly, method is electronic counting device inside dispensers, with data uploaded to a computer for direct feedback [23],[26]. This method also provides frequency rates for hand hygiene events.

Observation deserves its role in surveillance of compliance, because in addition to compliance rates it provides detailed information on the accuracy of the procedures performed. However, as observations were perceived as laborious a fair recommendation in equine hospitals would be to use purchase figures for longitudinal trends and apply point observation to monitor the accuracy of the procedures performed. Anecdotal evidence indicated that fewer observations were conducted during workload peaks, as the observer had to deal with ordinary tasks. A decreased number of observations during peaks could have biased the compliance figures, as high workload was a cause of reduced compliance according to the questionnaire. Nakamura *et al*. [18] also reported staff being too busy as a reason for less frequent hand washing and correlations between high work load activity and lower compliance rates have been reported in human healthcare too [21],[22]. To collect as correct data as possible, observations should be performed in different situations.

The exactness of observations is dependent on observer skills [24],[28]. To increase the objectivity of observations in human healthcare, use of trained, validated observers is considered the ‘gold standard’ in monitoring of hand hygiene accuracy and compliance [27]. However, a side-effect of this is the so-called Hawthorne effect, whereby behaviour is altered during observation if recognisable observers are used [25]. Therefore, we chose members of staff and provided them with brief training in the task. This solution might have caused another biases, e.g. that observers employed by the hospital at which the observations were performed unwittingly interpreted compliance permissively. Nevertheless, this has probably a minor influence on trends, provided that observations are interpreted in the same way over time.

In Sweden, a new regulation which came into force in April 2014 (SJVFS 2013:14) requires settings working with veterinary healthcare to have hygiene procedures in place. The regulation is aimed at preventing the spread of zoonoses, but also other infectious agents within animal healthcare. A national consensus statement or guidelines on minimum IC requirements would help hospitals or clinics plan their IC according to the upcoming regulation. The procedures currently used in veterinary IC have mainly been transferred from human medicine and for some procedures there is little scientific evidence of them being effective in equine medicine. Evidence-based practice would of course be ideal and the topic is wide open for research. There are species-dependent considerations; hence procedures formulated would have to be tested for efficacy [10]. Experts gathered at the third Havemeyer workshop on IC in equine populations agreed that surveillance, education and training are important tasks for IC in veterinary medicine [11].

## Conclusions

This study revealed some easily attainable improvements in IC worth considering for equine hospitals in Sweden: (i) formulating of traceable documented IC procedures, (ii) information and training of staff, especially hand hygiene and (iii) dialogue and involvement of staff in removing barriers to compliance. Furthermore, evaluation of the IC programme, as surveillance of compliance and monitoring of nosocomial infections was missing. For longitudinally monitoring of intra-hospital trends of compliance rates, purchases per patient seemed to suit the equine clinics best in daily work, and preferably complemented by direct observations.

Lack of association between education and measured compliance suggests various triggers of differing strength for compliance. Strong triggers must be identified in order to obtain long-term compliance, but is less studied within veterinary medicine, and in need of more research.

## Competing interests

The authors declare that they have no competing interests.

## Authors’ contributions

KB and UGA together initiated and designed the study, compiled, interpreted, evaluated and reported the results and formulated the conclusions. Both authors have read and approved the final version of the manuscript.
